# Diarrhea Caused by the Displacement of Percutaneous Endoscopic Gastrostomy Tube Tip Into the Duodenum: A Rare Case

**DOI:** 10.7759/cureus.40838

**Published:** 2023-06-22

**Authors:** Masaatsu Kuwahara

**Affiliations:** 1 Department of Emergency Medicine, Takarazuka City Hospital, Takarazuka, JPN

**Keywords:** percutaneous endoscopic gastrostomy, enteral nutrition, duodenum, tube displacement, diarrhea, percutaneous endoscopic gastrostomy (peg)

## Abstract

Percutaneous endoscopic gastrostomy (PEG) is a widely used procedure for patients with dysphagia and inadequate oral intake. Although PEG offers numerous benefits, complications can occur. Here, we present an unusual case of a 68-year-old woman who developed persistent diarrhea following a routine PEG tube exchange. Despite treatment attempts, her symptoms persisted, prompting further investigation. Abdominal computed tomography (CT) revealed the unexpected displacement of the PEG tube tip into the duodenum. Repositioning of the tube tip into the stomach resolved the diarrhea, and the patient was discharged without recurrence.

Diarrhea is a common gastrointestinal side effect in patients receiving enteral nutrition through a PEG tube, typically attributed to multiple factors. However, to our knowledge, this is the first reported case of diarrhea resulting from a PEG tube tip straying into the duodenum. The patient did not undergo any changes in enteral preparation or receive medications known to cause diarrhea. The identification of the tube misplacement was incidental during the CT scan, underscoring the importance of imaging studies in refractory cases.

While previous reports indicate no significant difference in diarrhea occurrence between duodenal and gastric feeding, our findings suggest that the presence of the PEG tube tip in the duodenum may contribute to diarrhea in some patients. This case highlights the potential role of CT imaging in diagnosing the cause of persistent diarrhea in PEG-fed individuals. Further accumulation of cases is necessary to establish the significance of duodenal tube placement as a cause of diarrhea during PEG procedures.

In conclusion, this case report emphasizes the importance of considering tube misplacement as a potential cause of refractory diarrhea in patients receiving enteral nutrition through a PEG tube. The use of abdominal CT imaging can be valuable in identifying such misplacements and guiding appropriate interventions. Further research is needed to validate these findings and explore the clinical implications for the management of PEG-related diarrhea.

## Introduction

Percutaneous endoscopic gastrostomy (PEG) plays a major role in the management of patients with inadequate voluntary oral intake, chronic neurological or mechanical dysphagia and bowel dysfunction, and serious illnesses. Despite the benefits and widespread use of PEG, some patients experience complications. Herein, we report an unusual case in which the tip of a PEG tube strayed into the duodenum, resulting in diarrhea that lasted for a month.

## Case presentation

**Figure 1:**
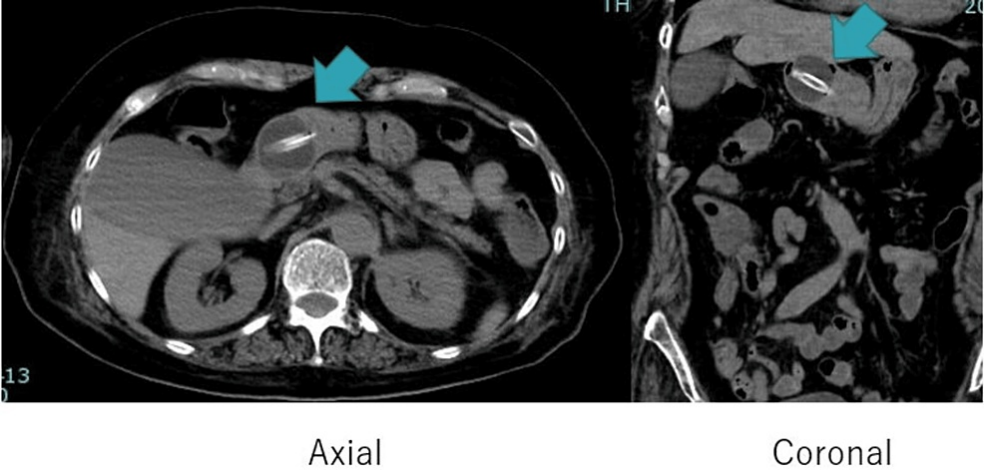
CT image at the time of arrival The tip of the percutaneous endoscopic gastrostomy tube has strayed into the duodenal bulb.

A 68-year-old woman had dysphagia due to a brain hemorrhage for which she had undergone PEG. She was under home care, and a visiting physician regularly performed PEG tube exchange. Routine PEG tube exchange took place one month prior to her visit to the emergency room. Subsequently, she experienced watery diarrhea (more than 10 times daily). The visiting physician administered loperamide and reduced the rate of enteral feedings, but to no avail. She was brought to our emergency department with low blood pressure and decreased urine output at home. Her blood pressure was maintained at 124/86 mmHg at arrival. Skin thrush was slightly decreased. A stool culture was performed to identify the cause of diarrhea, but it was negative, and the patient was not on any medications that could cause diarrhea (for example, laxatives, hyperosmotic drugs, and proton pump inhibitors). An abdominal computed tomography (CT) scan was performed to investigate the cause of diarrhea. It was suspected that the tip of the PEG tube, which was supposed to be implanted in the stomach, had strayed into the duodenum (Figure 1).

**Figure 2:**
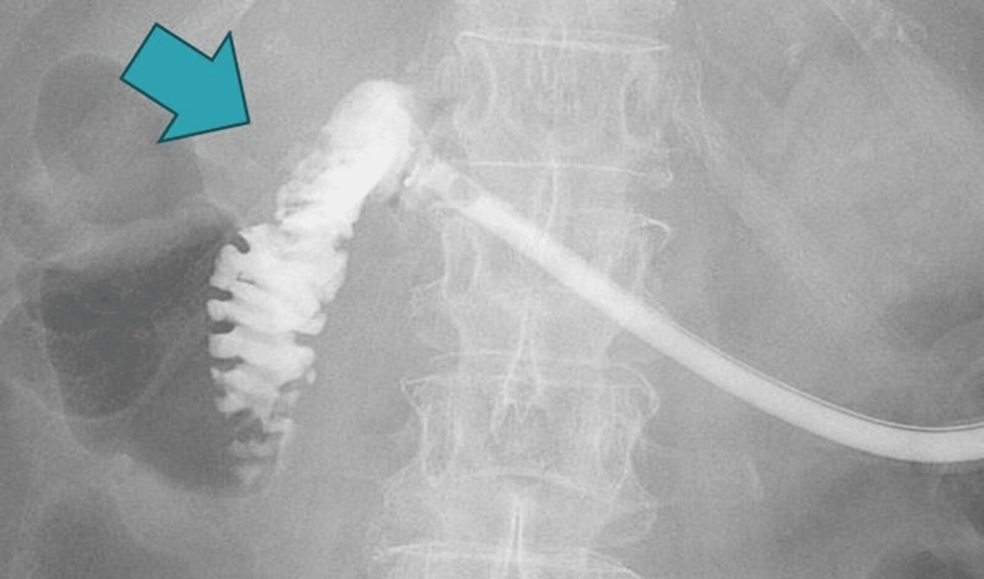
Contrast injection through the percutaneous endoscopic gastrostomy (PEG) tube Contrast examination through the tip of the PEG tube revealed no contrast in the stomach, but the presence of contrast in the duodenum.

The contrast agent was injected through the PEG tube in the fluoroscopy room. The stomach was not contrasted, but the contrast was injected into the duodenum (Figure 2).

**Figure 3:**
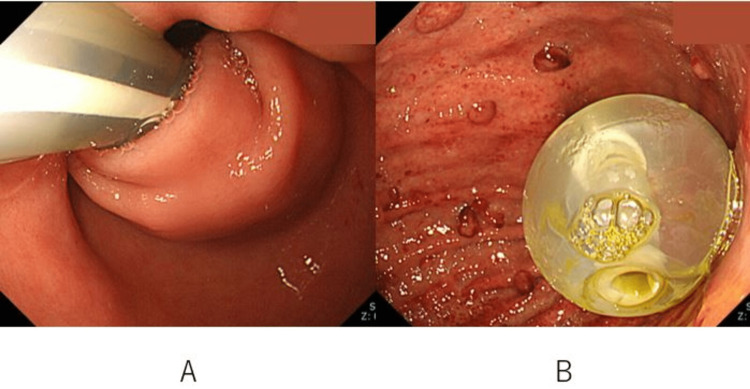
Upper gastrointestinal endoscopic findings A: The tip of the percutaneous endoscopic gastrostomy (PEG) tube has strayed behind the pyloric ring. B: The tip of the PEG tube is implanted in the stomach while confirming using an endoscope.

The tip of the PEG tube was repositioned in the stomach with endoscopic confirmation (Figure 3).

The patient was admitted to the hospital, and enteral feeding was resumed through the PEG tube. The patient was discharged without recurrence of diarrhea.

## Discussion

We encountered a rare case of diarrhea that lasted for a month because the tip of the PEG tube had strayed into the duodenum. Diarrhea is the most commonly reported gastrointestinal side effect in patients receiving enteral nutrition through a PEG tube [1-5], and there have been no reports of diarrhea caused by the tip of a PEG tube straying into the duodenum.

The causes of diarrhea in patients receiving enteral feeding are multifactorial, including the enteral products themselves, problems with the rate of administration, and the use of antibiotics and certain medications (magnesium, phosphate, antacids, and motility promoters) [2,5,6]. In the present case, the enteral preparation did not change and no antibiotics or specific medications were administered.

Abdominal CT was initially performed to detect intestinal edema. However, it was discovered by chance that the tip of the PEG tube had strayed into the duodenum, and the patient's diarrhea improved markedly after the tip was repositioned in the stomach.

A previous report showed no difference in the occurrence of diarrhea between patients who received enteral feeding through the duodenum and stomach [7]. However, if more cases, such as in the present report, are collected, the presence of the tip of the PEG tube in the duodenum may be a possible cause of diarrhea during PEG placement.

It was stated in a previous report that “The sudden influx of a hyperosmotic formula is likely to lead to abdominal cramping, hyperperistalsis and diarrhea since the jejunum relies on controlled delivery of isotonic substrates. An intrajejunal feeding is less physiological compared with an intragastric one. The ability of the stomach to distend and contain a large amount of food all at once is a great advantage compared to the limited distension capability of the jejunum” [8].

In the present case, too, enteral nutrition was bolus administered, and enteral nutrition exceeding the distending capacity of the duodenum was injected, causing diarrhea, and it is speculated that repositioning to the stomach may have improved diarrhea due to gastric distention.

One limitation of this study is that its applicability to other cases is not confirmed. Further accumulation of cases is required. In this case, the CT revealed the migration of the PEG tube, but if the physical findings had been carefully checked, it should have been possible to detect more than the location of the tube by marking the bumper, which was a point of reflection this time.

## Conclusions

Although various causes of diarrhea in patients receiving enteral nutrition through a PEG tube have been previously reported, our study suggests that the presence of the tip of the tube in the duodenum may be a cause of diarrhea in patients receiving enteral feeding through a PEG tube, and CT may be useful to detect the cause of refractory diarrhea in such patients.
